# Evaluating the effect of food components on the digestion of dietary nucleic acids in human gastric juice in vitro

**DOI:** 10.1002/fsn3.3599

**Published:** 2023-08-06

**Authors:** Yanfang Zhang, Jingyi Dong, Jingxian Chen, Xiaoming Pan

**Affiliations:** ^1^ School of Food Engineering Ludong University Yantai China; ^2^ Yantai Key Laboratory of Nanoscience and Technology for Prepared Food Yantai China; ^3^ Yantai Engineering Research Center of Green Food Processing and Quality Control Yantai China; ^4^ Institute of Bionanotechnology Ludong University Yantai China

**Keywords:** digestibility, food components, human gastric juice, microRNA, nucleic acids

## Abstract

Nucleic acids (NAs) were recently shown to be digested by pepsin in vitro; however, NAs digestion in human gastric juice in vivo is more complicated because of the complex gastric environment and ingestion of other food components. The purpose of this study was to investigate the digestibility of NAs in real human gastric juices after ingestion of other food components. As a result, DNA digestion was not affected when carbohydrates, proteins, and metal elements were ingested within the recommended dietary intake levels. Separately, protein exerted an inhibitory effect on DNA digestion when the mass ratio of protein:DNA was greater than 40:1. DNA exists in the nucleoprotein, which is closer to the state of DNA in real food, and was digested efficiently in human gastric juice. Meanwhile, DNA digestion was rarely affected even when the concentrations of monovalent ion (Na^+^) and divalent ions (Mg^2+^) were as high as 500 and 100 mM, respectively, and high concentration of Mg^2+^ ranged from 20 to 100 mM accelerated the digestion. In particular, short‐stranded DNA (<100 nt) and miRNAs (19 ~ 25 nt) were not obviously degraded in human gastric juice. In conclusion, dietary NAs were digested efficiently and were not affected by other food components in human gastric juice, which may facilitate further digestion and utilization of DNA in the intestinal tract.

## Graphical abstract

Long‐stranded DNA was degraded efficiently in human gastric juice in vitro. Food components in the recommended dietary range levels had little effect on DNA digestion, and high concentration of divalent ion (e.g., Mg^2+^) could accelerate DNA digestion. Short‐stranded DNA and miRNAs could not be degraded in human gastric juice in vitro.

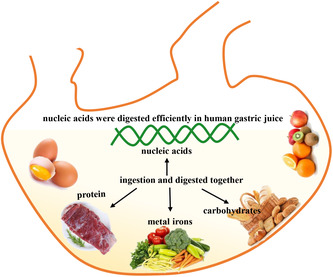

## 1 INTRODUCTION

Nucleic acids (NAs), including deoxyribonucleic acids (DNAs) and ribonucleic acids (RNAs), have been ignored as dietary ingredients for many years; actually, NAs account for 15% of the dry weight of food ingredients and are second only to carbohydrates and proteins (Gil et al., 2007). The traditional view holds that dietary NAs are digested by nucleases in the intestine and absorbed in the intestinal tract in the form of nucleotides and their related metabolic products (Carver & Allan, 1995). The ingested NAs were shown to play key roles in many biological processes, for example, repairing intestinal injury and other damaged cells, providing a nutritional supplement for infant formula milk powder, and maintaining the cellular immune response (Aird & Zhang, 2015; Che et al., 2016; Singhal et al., 2010; Zhang et al., 2020).

However, in terms of the fate of exogenous NAs after food ingestion, the traditional views have been challenged by many studies in recent years. As reported by (Schubbert et al., 1998) DNA oligonucleotide fragments in the range of 200 to 500 bp were detected in mouse feces and blood respectively after feeding of M13 genes. (Hohlweg & Doerfler, 2001) reported that 1600 bp DNA fragments from soybean could be detected in mice intestinal after oral ingestion for 49 h. Another study found that the absorbed amount and utilization of oligonucleotide fragments were closely related to its sequence specificity, and the “survived” dietary DNA fragments were absorbed into animal cells by the way of active transport (Basner‐Tschakarjan et al., 2004; Lehmann & Sczakiel, 2005). These studies have shown that some NAs prevented degradation in the mammalian gastrointestinal (GI) tract, including the strong acidic environment of the stomach and the complicated condition of the intestinal tract, where nucleases and associated microbiota are abundant (O'Neill et al., 2011; Philips et al., 2022). Not only DNA, the latest report showed that dietary microRNAs (miRNAs) resist damage caused by the acidic environment of gastric juices and are absorbed in the stomach (Chen et al., 2021). Therefore, exogenous NAs have the opportunity to enter the circulatory system of animals or the humans, and to exert physiological function or regulate gene expression (Sanchita et al., 2018). Overall, these studies presented a conclusion that food‐sourced NAs might remain stable in organisms and regulate physiological processes, which is still being debated.

The digestibility of NAs in the GI tract must be evaluated to address the problems and controversies described above. As shown in our previous study, naked DNA was digested well by pepsin in vitro (Liu et al., 2015). However, on the one hand, the real human gastric juice component is more complicated, and digestion is a dynamic process. On the other hand, DNA in food is tightly packed by histones as a nucleoprotein (NP) complex that is taken up together with other food components (Bonner et al., 1986), thus preventing direct binding between pepsin and NAs. Therefore, compared with naked DNA digestion by pepsin, digestion of dietary NAs in the stomach is more complicated.

Based on the facts described above, we hypothesized that food components ingested together with NAs would affect NA digestion in the stomach. As a method to more closely mimic the NA digestion state in the stomach, common food components, including proteins, carbohydrates, and metal ions, were employed to evaluate their effects on DNA digestion. Various concentrations of food components were used to simulate the varying dietary intake levels. The digestion of NP extracted from *Corvina* was also investigated. Moreover, several human gastric juice samples were employed, which were viewed as a practical gastric environment in vivo. Specifically, the digestion of short‐stranded DNA (<100 nt) and three different miRNAs in human gastric juice were also investigated briefly. This study is important for understanding the fate of dietary NAs in the digestive tract and is useful for the study of NA nutrition. In addition, we hope this study will provide some useful information for the evaluation of oral gene therapy drugs.

## 2 MATERIALS AND METHODS

### 2.1 Materials

Lumbda DNA (λDNA) (48.5 kb) was purchased from Thermo Fisher Scientific (MA, USA) with concentration of 300 μg/mL. The storage buffer for λDNA was 10 mM Tris–HCl (pH 7.6) containing 1 mM EDTA. Hemoglobin (HB) (H2625) was purchased from Sigma Co., Ltd. Salmon sperm DNA (D1626) purchased from Sigma was dissolved in ddH_2_O at a concentration of 300 ng/μL. Analytical pure starch, NaCl and MgCl_2_ were purchased from Beijing Solarbio Science & Technology Co., Ltd. All the carbohydrates were dissolved in ddH_2_O with concentration of 40 mg/mL for storage. miR168a, miR16, miR206, and short‐stranded DNA were synthesized by Genscript Biotech and were dissolved in sterile Milli‐Q water (ddH_2_O) at a 10 μM concentration for use as stock solutions.

### 2.2 Assay of pepsin activity in human gastric juice

Human gastric juices were provided by Department of Biochemistry and Molecular Biology, Qingdao University Medical College. Activity of pepsin in human gastric juice was evaluated by the method of (Johnston et al., 2007) with a slight modification using hemoglobin (HB) as the substrate. A volume of 50 μL human gastric juice was mixed with 250 μL (2.5 mg) of 37°C equilibrated HB substrate (pH 3.0), and incubated at 37°C for 10 min. Enzyme reaction was terminated by adding 500 μL of 5% (w/v) TCA, mixed by swirling and incubated at 37°C for 5 min. The reaction mixture was centrifuged at a 12,000 r/min for 10 min, and the absorbance of oligopeptides content in the supernatant was measured at 280 nm. One unit of activity was defined as the increase of 0.001 in absorbance at 280 nm per minute. The blank was carried out in the same manner, except that the enzyme was added after the addition of 5% (w/v) TCA.

### 2.3 Digestion of λDNA in human gastric juice

Gastric juices from eight healthy individuals were collected, and then pH values and pepsin activity were measured. A volume of 2 μL λDNA (300 ng/μL) was added into 10 μL of human gastric juice, the final reaction volume was 20 μL (including 8 μL of ddH_2_O), and reactions were incubated at 37°C for 5 h. After that, samples were extracted by phenol–chloroform–isopentanol (25:24:1/v:v:v) method to remove pepsin in gastric juice, and a volume of 10 μL supernatant was used for electrophoresis on a 1% agarose gel.

### 2.4 Digestion of λDNA in human gastric juice along with food component

#### 2.4.1 Digestion of DNA along with multiple food components

The effects of food components on the digestion of DNA were evaluated by adding 2 μL starch, 2 μL HB, 2 μL NaCl, and 2 μL of ddH_2_O to 10 μL of gastric juice along with 2 μL λDNA. The mass ratio of starch: HB: DNA was 80:40:1 (where 1 is the amount of λDNA), according to World Health Organization's recommendation of daily nutrient intake. Besides, a mass of 150 mM NaCl was added to mimic the dietary intake of salts. The mixture was digested at 37°C for 5 h and then analyzed in Section 2.3.

#### 2.4.2 Effect of protein on the digestion of λDNA in human gastric juice

Hemoglobin (HB) (Mw 67,000 Da) was used to evaluate the influence of protein on DNA digestion. HB (24 mg/mL) was dissolved in 1 mM HCl before use. Aliquot of 2 μL HB and 2 μL λDNA was digested in 10 μL of human gastric juice, and 6 μL of ddH_2_O was added to a total volume of 20 μL. The mass ratios of HB:λDNA were designated as 10:1, 20:1, 30:1, 40:1, 60:1, 80:1, 200:1, and 400:1, respectively. All the reactions were carried out at 37°C for 5 h and then analyzed in Section 2.3.

#### 2.4.3 Digestion of nuclear protein (NP) in human gastric juice

Corvina was obtained from a local market, and the spermary was excised as soon as the fish was killed. The nuclear protein was extracted based on the protocol of (Bonner et al., 1986), and the measured protein content was approximately 37% using a kit (PC0020) purchased from Solarbio.

Aliquot of 4 μL NP (30 mg/mL) was digested in 10 μL of human gastric juice, and a volume of 6 μL ddH_2_O was added to a final volume of 20 μL. The mixtures were incubated at 37°C for 5 h. After reaction, pepsin in human gastric juice and protein in NP were removed and then analyzed in Section 2.3.

#### 2.4.4 Effect of metal ions on the digestion of DNA in human gastric juice

Aliquot of 2 μL NaCl (or MgCl_2_) at different concentrations was mixed together with 2 μL λDNA, 10 μL human gastric juice, and 6 μL ddH_2_O to a total volume of 20 μL, the final concentrations of NaCl were 20, 50, 100, 150, 200, 300, 400, and 500 mM, respectively. For MgCl_2_, the final concentrations were 2, 5, 10, 20, 50, and 100 mM, respectively. The mixtures were incubated at 37°C for 5 h and then analyzed in Section 2.3.

### 2.5 Digestion of short‐stranded DNA and microRNA in human gastric juice

Aliquot of 10 μL of human gastric juice was added into 8 μL of sterilized water (RNase free), and then 2 μL miRNA (or short DNA) was added, the final concentration of miRNA (or short DNA) was 2 μM. The mixtures were incubated at 37°C for 5 h and then extracted by phenol–chloroform–isopentanol (25:24:1/v:v:v) method to remove protein in gastric juice, the supernatant of 10 μL was used for electrophoresis on a 15% denatured polyacrylamide gel.

## 3 RESULTS AND DISCUSSION

### 3.1 Digestion of λ DNA in human gastric juice

Practical human gastric juices were employed to better evaluate NA digestion in the GI tract, which was regarded as the real gastric environment in vivo. Eight human gastric juice samples collected from healthy individuals were selected for further study. The basic information on these eight human gastric juice samples is provided in Table 1. The pH values ranged from 1.3 to 2.9, all of which were within the normal pH range, and pepsin activity in human gastric juice was measured (Table 1). In addition, both extracted salmon sperm DNA and bacteriophage λ (λ DNA) were used to mimic the long dietary DNA digestion by human gastric juices. Considering that λ DNA is a kind of double‐stranded DNA with a fixed length of 48.5 kb, therefore, it was easy to be observed after degradation. Therefore, λ DNA was used to study the digestibility of NAs in human gastric juice in the following study. Meanwhile, digestion results of extracted salmon sperm DNA by human gastric juices were shown in Figure S1, in which both the original DNA and the degradation products were dispersive bands.

**TABLE 1:** Information of eight human gastric juices.

Gastric juice No.	1	2	3	4	5	6	7	8
pH value	2.6	2.9	2.3	2.8	1.7	2.9	1.6	1.3
Enzyme activity (U/mL)	830	880	960	1080	1510	1070	990	2110

**FIGURE 1:**
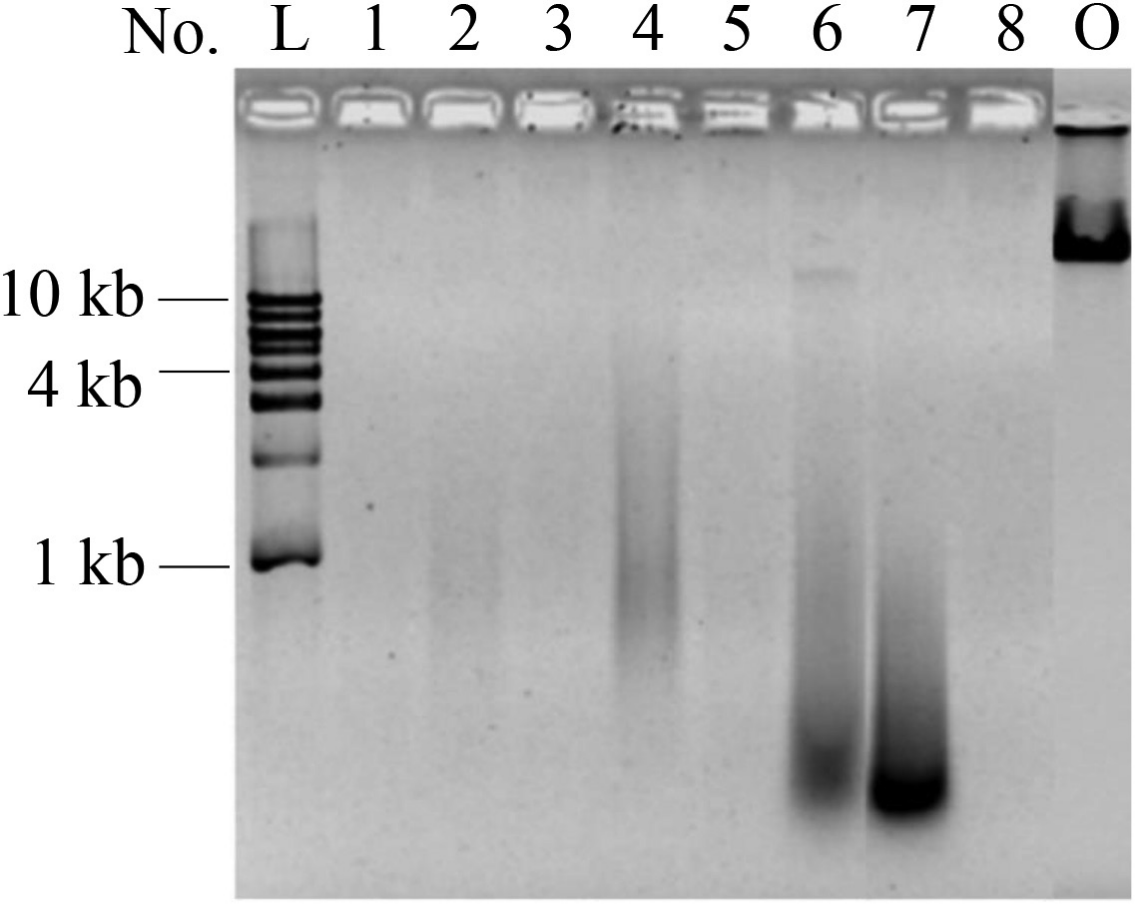
Digestion of λ DNA in human gastric juice samples. L: DNA ladder; lane 1 to lane 8: λ DNA was digested in eight different human gastric juice samples; O: original λ DNA. All reactions were incubated at 37°C for 5 h.

As shown in Figure 1, almost all λ DNA was destroyed, and most fragments were shorter than 1000 base pairs, suggesting that naked DNA was digested efficiently in the stomach. Obviously, DNA degradation differed among gastric juice samples, and this phenomenon was mainly attributed to individual differences. As shown in Table 1, the pH value and enzyme activity of No. 1, No. 2, No. 4, and No. 6 gastric juices were about the same; however, DNA degradation in No. 6 gastric juice was different from other three (Figure 1, lane 1, 2, 4, and 6). It was apparent that No. 6 and No. 7 gastric juices were different in pH values (Table 1); however, DNA degradation was about the same (Figure 1, lane 6, and lane 7). Therefore, individual difference among gastric juices was an important factor which influence DNA digestion. The excellent DNA digestion ability of human gastric juices was excited, and the result was consistent with our previous study showing that DNA was digested by pepsin under acidic conditions in vitro (Liu et al., 2015). Unfortunately, we were not able to simulate the dynamic process of DNA digestion, under which conditions the digestion efficiency would be much higher because of sufficient contact between DNA and pepsin. One may wonder which factor was the key to DNA digestion, pepsin, or the acidic environment. DNA digestion in simulated gastric juices in vitro was conducted to answer this question. Pepsin mainly contributed to NA digestion; for those reactions lacking pepsin, NA degradation was not observed (Figure S2).

### 3.2 Digestion of DNA in human gastric juice along with food components

Three main nutritional elements (starch, protein, and metal ions) were added to the digestion systems to better mimic the digestion of dietary DNA in the stomach. According to Chinese Dietary Reference Intakes, the recommended intakes of carbohydrates and protein were 120 and 55–65 g/day, respectively. The suggested intake of dietary NAs should be less than 1.5 g/day, as recommended by the World Health Organization (WHO). Thus, the appropriate mass ratio of carbohydrates:protein:λ DNA was calculated to be 80:40:1 (120:60:1.5 g/day). For metal ions, the most commonly used flavor of NaCl was investigated first, and the intake concentration in each meal was calculated to be 200 mM, as reported by (Zhang, Wang, et al., 2016).

**FIGURE 2:**
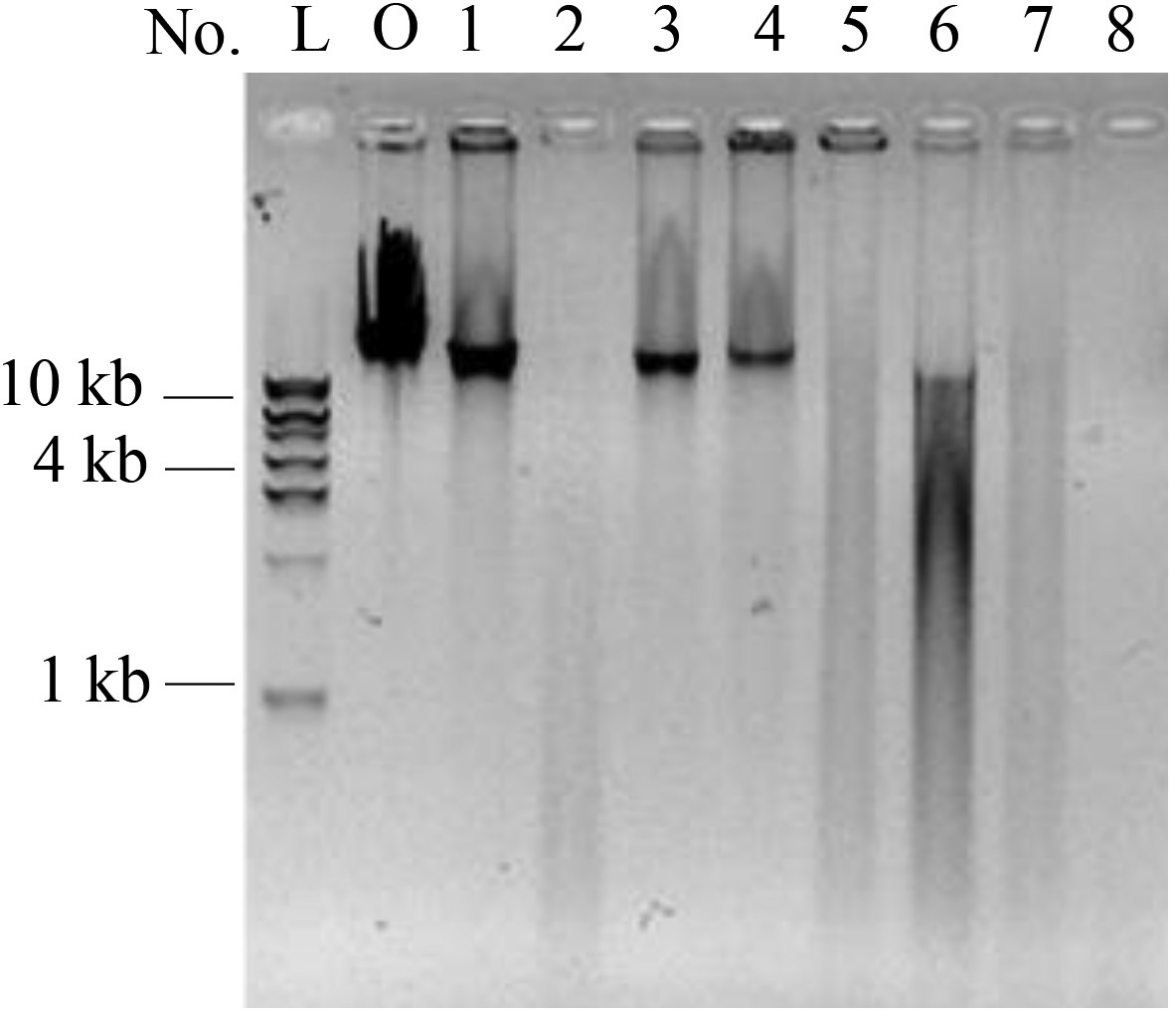
Digestion of DNA in human gastric juice along with other food components. O: original λ DNA. L: DNA ladder; lane 1 to lane 8: λ DNA was digested together with other food components. All reactions were incubated at 37°C for 5 h.

As shown in Figure 2, five of eight λ DNA samples were degraded into short fragments in human gastric juices when starch, protein, and NaCl were added simultaneously (Figure 2, Lane 2, and Lane 5 to Lane 8). Surprisingly, the degradation for these five DNA samples was approximately the same as that of the naked DNA in Figure 1, indicating that digestion of dietary DNA was less influenced by other food ingredients within a reasonable dietary intake range. However, DNA in the other three human gastric juices was only slightly degraded (Figure 2, Lane 1, Lane 3, and Lane 4), which was completely different from the naked DNA digestion (Figure 1, Lane 1, Lane 3, and Lane 4). Several possible reasons for the significant difference were considered. Pepsin activity might be inhibited by NaCl, as reported by (Klomklao et al., 2007). However, the relative activity of pepsin was approximately 84%, even when the NaCl concentration was as high as 600 mM (Zhang, Wang, et al., 2016); therefore, the inactivation of pepsin activity by NaCl might not be the main factor inhibiting DNA digestion. Second, a high concentration of food ingredients might prevent contact between DNA and pepsin; in particular, protein, as the optimum substrate for pepsin, might compete with DNA. To further verify the above two possibilities, the effects of protein and metal ions on DNA digestion were subsequently investigated. However, regardless of the true reason for the inhibited digestion, we cannot neglect the effects of individual differences, because human gastric juice contains components other than pepsin and hydrochloric acid, such as mucous, inorganic salt, and intrinsic factors (Brodkorb et al., 2019). All these factors might affect DNA digestion.

### 3.3 Effect of protein on DNA digestion in human gastric juice

**FIGURE 3:**
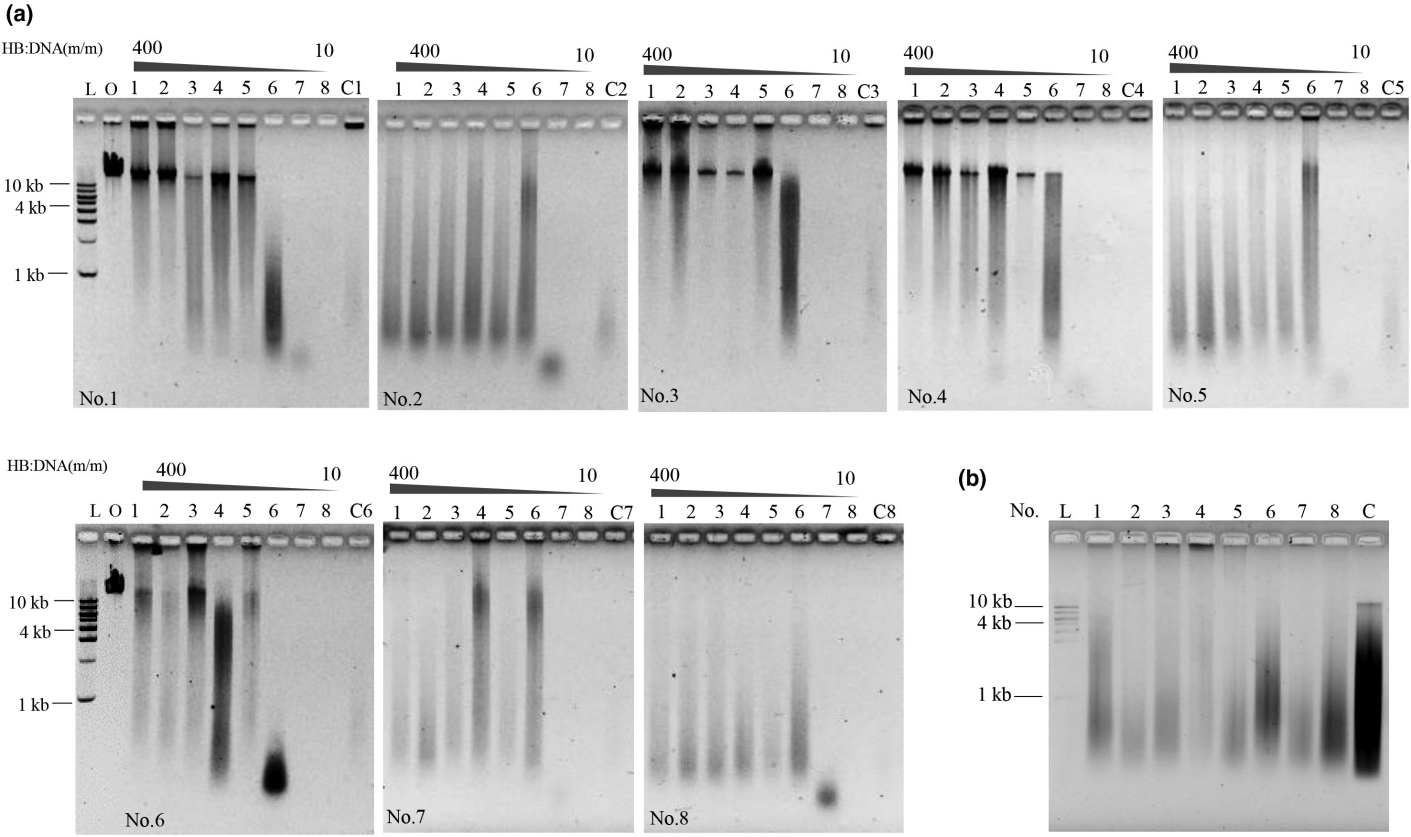
Effect of protein on λ DNA digestion in human gastric juice samples. (a) λ DNA was digested together with protein. L: DNA ladder; O: original λ DNA. The mass ratio of HB:DNA was 400:1, 200:1; 80:1; 60:1; 40:1; 30:1, 20:1, and 10:1 by sequence; C1 to C8 were the digestion of λ DNA without addition of protein. The image of the DNA ladder in each figure was similar; therefore, the lanes of ‘L’ were removed apart the first figure. (b) Nucleoprotein was digested in human gastric juice. C: Original Nucleoprotein. All reactions were incubated at 37°C for 5 h.

Protein is the optimal substrate for pepsin, which means that protein might function as a competitive inhibitor during DNA digestion. Therefore, HB as one of the optimal substrates for pepsin was employed to investigate the effect of protein on DNA digestion in human gastric juice. The mass ratio of protein:DNA ranged from 10:1 to 400:1. As shown in Figure 3a, when the mass ratio of protein:DNA was lower than 40:1, protein did not affect DNA digestion (Figure 3a, Lanes 6–8 in samples No. 1 to No. 8). When the mass ratio of protein:DNA was greater than 40:1, DNA digestion was inhibited compared with naked DNA (Figure 3a, Lanes 1–5 in samples No. 1 to No. 8), suggesting that DNA digestion in human gastric juice depended on the protein concentration. The inhibitory effect of a high concentration of protein on DNA digestion might be explained by the fact that protein is the optimal substrate of pepsin, and the affinity between pepsin and protein is higher than that of DNA. The catalytic efficiency of pepsin on DNA was approximately 10,000 times lower than that on protein, as reported in our previous study (Liu et al., 2015). Therefore, when the protein concentration was higher than that of DNA (≥40:1), pepsin would be occupied by protein and fail to be dock with DNA; thus, DNA digestion would be inhibited.

Most DNA in food exists in the form of NP and is not present in the naked form (Craigie, 2018). The digestion of NP extracted from *Corvina* spermary was investigated to further elucidate the effect of protein on DNA digestion in food. The results are shown in Figure 3b; NPs were digested efficiently in eight human gastric juice samples compared with the equal mass of NPs without digestion. Despite the fact that DNA in NP was wrapped tightly by an abundance of proteins, it was degraded well. This might be due to the reason that protein as the optimal substrate for pepsin was degraded quickly, and then DNA was exposed and degraded. Based on these results, the effect of protein (regardless of whether the protein was in the diet or NP) on DNA digestion was weak, and dietary DNA was digested efficiently in human gastric juice even when a large amount of protein was ingested together with DNA.

### 3.4 Effect of metal ions on DNA digestion in human gastric juice

**FIGURE 4:**
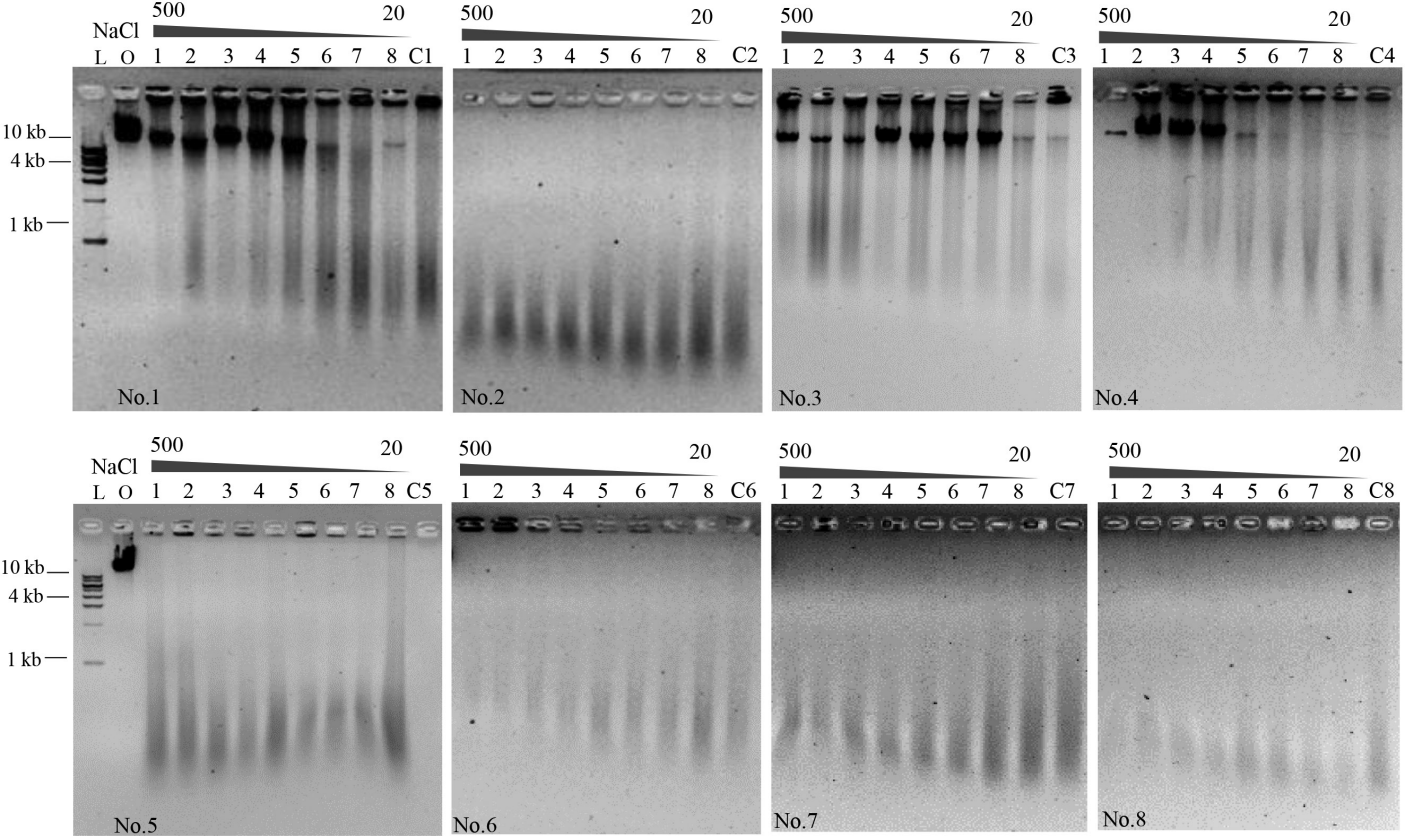
Effect of NaCl on λ DNA digestion in human gastric juice samples. L: DNA ladder; O: original λ DNA. Concentrations of NaCl were 500, 400, 300, 200, 150, 50, and 20 mM by sequence. C1 to C8 were the digestion of λ DNA without addition of any metal ions. The image of the DNA ladder in each figure was similar; therefore, the lanes of ‘L' were removed apart the first figure. All reactions were incubated at 37°C for 5 h.

**FIGURE 5:**
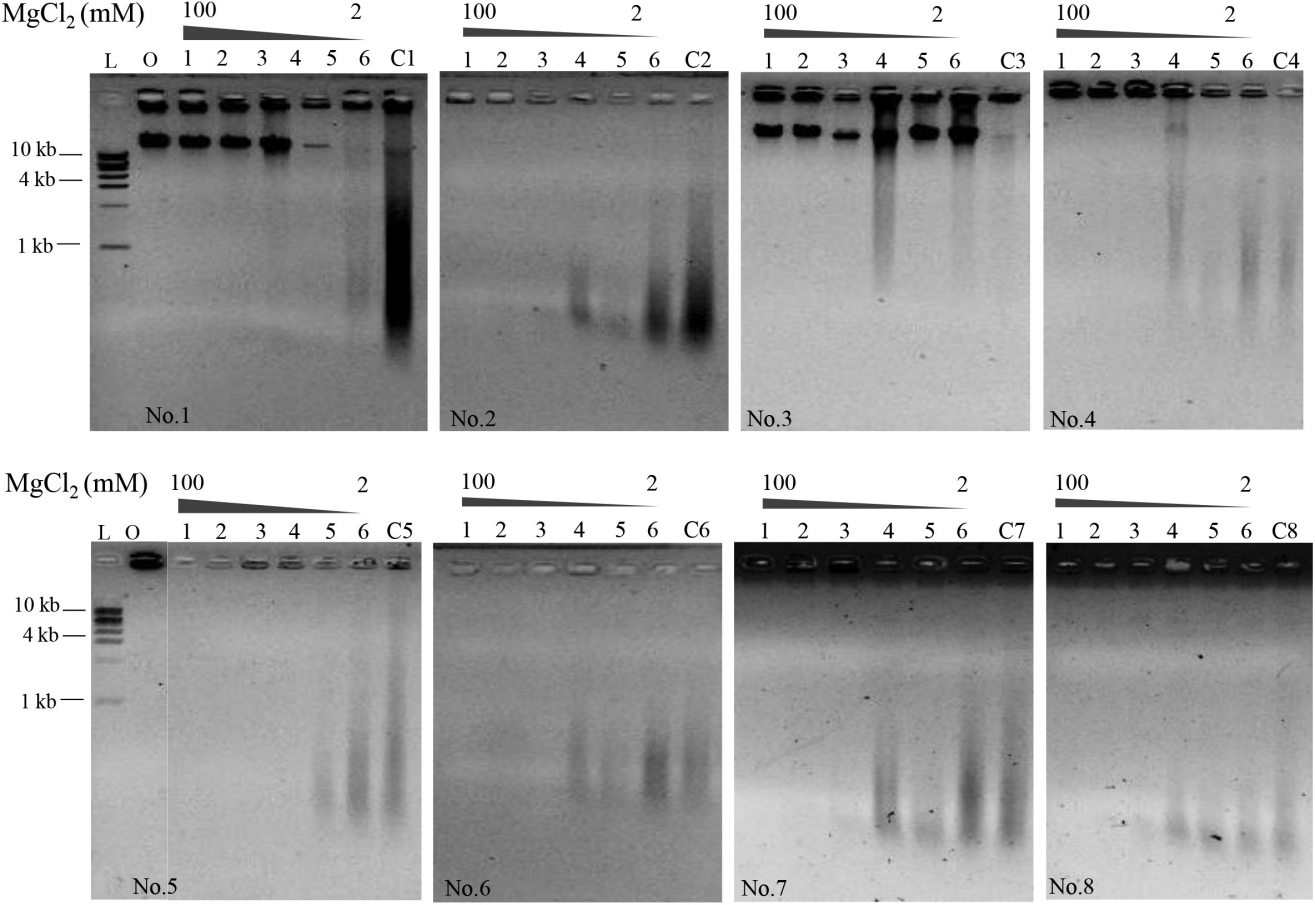
Effect of MgCl_2_ on λ DNA digestion in human gastric juices. L: DNA ladder; O: original λ DNA. Concentrations of MgCl_2_ were 100, 50, 20, 10, 5, and 2 mM. C1 to C8 were the digestion of λ DNA without addition of any metal ions. The image of the DNA ladder in each figure was similar; therefore, the lanes of ‘L' were removed apart the first figure. All reactions were incubated at 37°C for 5 h.

As reported by (Klomklao et al., 2007) the digesting activity of pepsin toward protein is substantially affected by metal ions. However, metal elements are inevitably ingested in the daily diet. NaCl, the most popular flavoring that is widely added to food, is used as an example. Other metal ions, such as potassium, calcium, and magnesium, exist abundantly in many types of food and are considered essential elements for human health. Therefore, we investigated the effects of the monovalent cation Na^+^ and divalent cation Mg^2+^ on DNA digestion, and the results are shown in Figures 4 and 5. When the concentration of NaCl increased from 20 to 500 mM, no obvious effect on DNA digestion was observed even when the concentration was as high as 500 mM in human gastric juice samples No. 2 and No. 5 to No. 8 (Figure 4, Lanes 1–8). Similar results were observed when divalent Mg^2+^ was added; the effect of Mg^2+^ on DNA digestion could be neglected when the concentration was between 2 and 10 mM (Figure 5, Lanes 4–6 in samples No. 2 and No. 5 to No. 8). In particular, when the concentration of Mg^2+^ was greater than 10 mM, DNA digestion was accelerated remarkably, and λ DNA was degraded into fragments much shorter than 1 kb; therefore, no digestion products were observed on the agarose gel (Figure 5, Lanes 1–3 in samples No. 2 and No. 5 to No. 8). These above results indicated that DNA digestion in human gastric juice was minimally affected by metal ions; instead, a high concentration of divalent cations significantly promoted DNA digestion.

Compared with DNA digestion in simulated gastric juice in vitro, we observed increased tolerability of DNA digestion in human gastric juice. For example, NaCl exerted a significant inhibitory effect at a concentration of 320 mM when DNA was digested in a simulated gastric juice in vitro, and the inhibitory concentration of MgCl_2_ was 50 mM (Zhang, Wang, et al., 2016). In contrast, DNA digestion in human gastric juice was unaffected by the two concentrations of ions described above (Figure 4, Lane 3 and Figure 5, Lane 2). The difference might be caused by the complicated components of human gastric juice, in which pepsin was not as sensitive to variations in the metal ion concentration, although the relative pepsin activity was only reduced by 13% in the presence of a high NaCl concentration (600 mM). NaCl is a common flavoring used in food, and its ingestion amount is closely related to individual eating habits. To investigate whether different NaCl intakes could inhibit DNA digestion, we calculated the average daily intake of NaCl, according to the reports that people should ingest a mass of 6–15 g NaCl through meals one day (Ge, 2011; Sacks et al., 2001), our gastric residual volume (GRV) is approximately 50–250 mL, and the stomach expands approximately 1000 mL during meal ingestion due to endogenous secretions (Liu et al., 2022; Maltby et al., 2004). Thus, we calculated the concentration of NaCl in each meal to be 34–85 mM. With the addition of normal saline at approximately 150 mM, the NaCl concentration in the stomach due to the diet was approximately 190–240 mM. Therefore, DNA digestion in human gastric juice would not be affected even when double the recommended amount of NaCl was ingested.

However, the inhibitory effects of NaCl and MgCl_2_ on DNA digestion were significant in human gastric juice samples No. 1, No. 3, and No. 4 (Figures 4 and 5). Considering that the inhibitory effect of protein on DNA digestion was also observed when the recommended protein:DNA mass ratio was 40:1 in these three gastric juices (Figure 3a), we inferred that complicated components and individual differences in human gastric juices were the main reasons for the inhibitory effects.

### 3.5 Digestion of short‐stranded DNA and microRNA in human gastric juice

**FIGURE 6:**
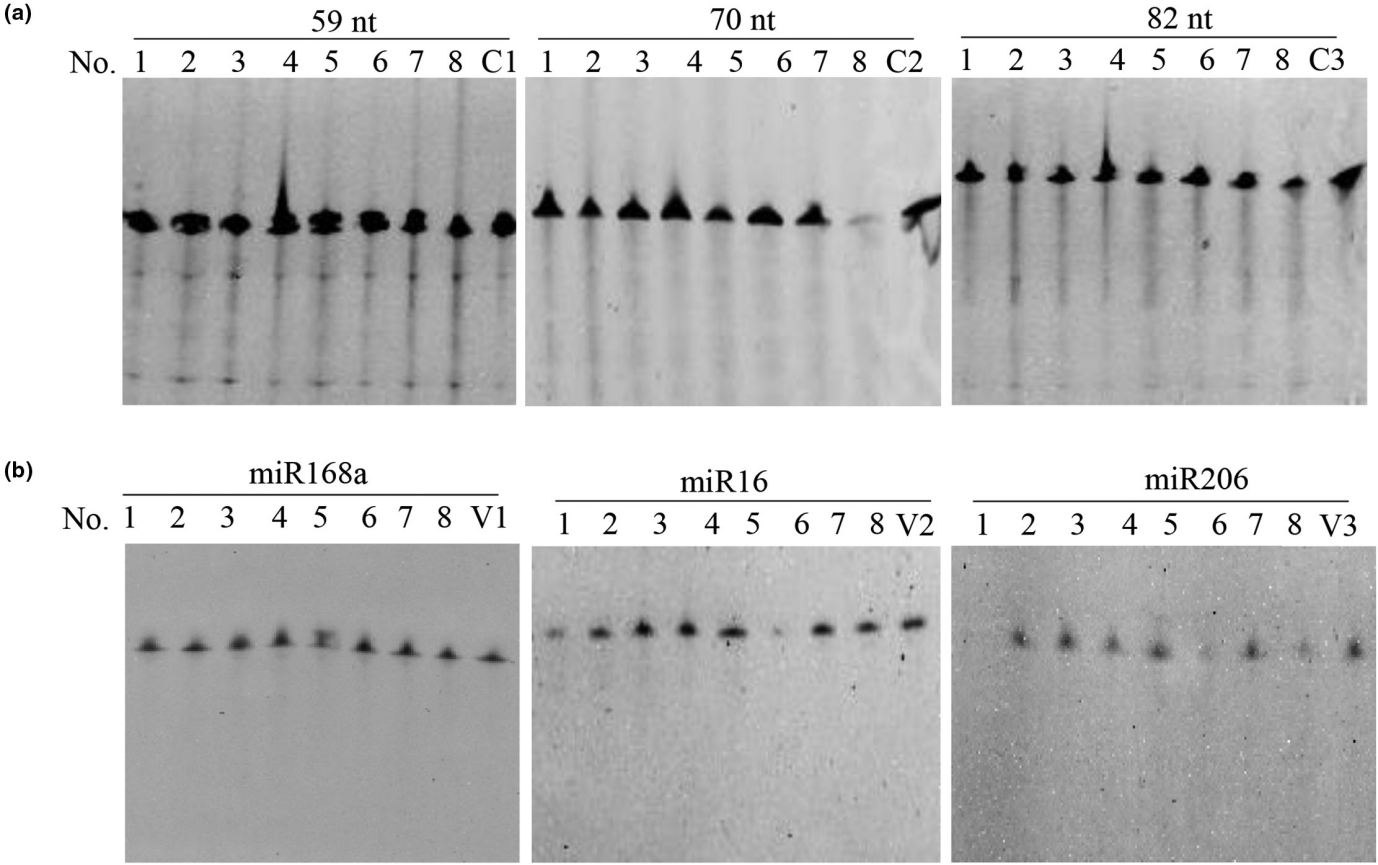
Digestion of short DNA (a) and miRNA (b) in eight human gastric juice samples. Lane 1–8: DNA/miRNA was incubated in human gastric juices. C1, C2, and C3: FITC‐labeled short DNA was incubated at pH 3.0 HCl solution. V1, V2, V3: miRNA was incubated at pH 3.0 HCl solution. All reactions were incubated at 37°C for 5 h.

Long‐stranded DNA and RNA (longer than 100 bp/nt) can be digested by pepsin (Liu et al., 2015). A reasonable explanation is that long‐stranded nucleic acids provide more combined sites and cleavage sites for pepsin and thus are more readily digested. However, part of the DNA was inevitably destroyed into short fragments during food processing (James et al., 2021), and the typical length of miRNAs is 19–24 nt. Therefore, are short‐stranded DNA (ssDNA) and miRNAs digested in human gastric juice? Here, ssDNA with lengths less than 100 nucleotides and several miRNAs were investigated briefly, and the sequence information is shown in Table 2. As shown in Figure 6a, unlike long‐stranded λ DNA, short‐stranded DNA of 59, 70, and 82 nt was still intact after an incubation for 5 h, indicating that ssDNA was more stable in human gastric juice. However, ssDNA (<100 nt), which was used in the above study, could be digested by pepsin in simulated gastric juice (Zhang, Li, et al., 2016). This difference might be attributed to the different digestive circumstances between a practical gastric juice sample and a simulated sample. ssDNA is more stable in practical human gastric juice, where the components are more complex. Our subsequent study showed that ssDNA was absorbed into the Caco‐2 cells (Figure S3); it is possible that some of ssDNA which is hard to be digested in gastric juice will survive in the stomach and be absorbed in intestinal epithelial cells, and thus may play biological significance.

**TABLE 2:** Sequences of short ssDNA and miRNA used in this study.

Name	Sequence (5′ → 3′)	Length/nt
FITC‐82	GTCCTAATCGTCTCGCTCCATACATCCGCCACGATGTCTCAAGAACTTCACCACCGGATTGATGCCGATGTGATCTTCTTGT	82
FITC‐70	TCGCTCCATACATCCGCCACGATGTCTCAAGAACTTCACCACCGGATTGATGCCGATGTGATCTTCTTGT	70
FITC‐59	ATCCGCCACGATGTCTCAAGAACTTCACCACCGGATTGATGCCGATGTGATCTTCTTGT	59
mi168a	UCGCUUGGUGCAGAUCGGGAC	21
mi16	UAGCAGCACGUAAAUAUUGGCG	22
miR206	UGGAAUGUAAGGAAGUGUGUGG	22

The results of miRNA digestion are shown in Figure 6b. Unexpectedly, miRNAs, which are presumed to be more fragile, survived the extreme pH and digestive enzymes in gastric juice, although miR206 was degraded in human gastric juice sample No. 1 (Figure 6b, Lane 1 for miR206). This result was consistent with the study by Chen et al., who showed that plant miRNAs survive the acidic conditions of gastric juice and are absorbed directly into the stomach (Lehmann & Sczakiel, 2005). On the one hand, the stability of miRNAs in gastric juice might be explained by the result of our previous study showing that the optimum DNA substrates for pepsin are long double‐stranded DNA and purine‐rich DNA (Liu et al., 2022). Notably, miRNAs are single‐stranded, the first nucleotide generally starts with the base U, and most of them have a lower purine content. On the other hand, the enzymatic activity of RNase was inactivated under acidic conditions to ensure the stability of miRNAs in the stomach. Therefore, a reasonable hypothesis is that miRNAs were more stable than other NAs in human gastric juice.

## 4 CONCLUSIONS

In summary, our study demonstrated that dietary NAs were digested efficiently in human gastric juice during regular diet, and other food components, including protein, metal ions, and carbohydrates at the recommended daily intake levels, had little effect on DNA digestion. Especially, a high concentration of Mg^2+^ accelerated digestion, and a high protein concentration increased DNA tolerance to the human gastric environment. The differential effects of magnesium ions and protein on NA digestion indicate that individual eating habits are important for the digestion of dietary NAs. For those people who like to eat food rich in protein or magnesium ions, NA digestion may be inhibited or accelerated accordingly.

Additionally, our study was significant for further perfection of NA digestion pathway in vivo. Digestion of long‐stranded NAs in the stomach can facilitate NA digestion and absorption in the intestinal tract, and thus, dietary NAs may play physiological functions more effectively in vivo. Specifically, short‐stranded DNA and miRNAs survived pepsin digestion and acidic environments, and the good stability of ssDNA and miRNAs in human gastric juice was significant. As reported, NAs that are absorbed must escape nucleases in the first barrier of gastrointestinal tract, cellular compartments, and in the bloodstream (Zhang et al., 2012). Therefore, we hope our results will provide useful information for evaluating the release and digestion of oral gene therapy drugs in GI tract. Despite the aforementioned strengths, a more complicated digestion system was still lacking in this study. For example, the food ingredient of lipids was neglected, and although practical gastric juices were used, the dynamic digestion could not be simulated. Therefore, future studies are needed to more closely mimic real digestion in vivo, and the mechanism by which the body absorbs the surviving exogenous NAs should be studied in detail.

## AUTHOR CONTRIBUTIONS

**Yanfang Zhang:** Funding acquisition (lead); writing – original draft (lead). **Jingyi Dong:** Formal analysis (equal). **Jingxian Chen:** Project administration (equal); software (equal). **Xiaoming Pan:** Funding acquisition (supporting); writing – review and editing (supporting).

## CONFLICT OF INTEREST STATEMENT

The authors declare that they have no conflict of interest regarding the publication of this.

## Supporting information


Figure S1.
Figure S2.
Figure S3.

## Data Availability

The data that support the findings of this study are available from the corresponding author upon reasonable request.
