# Supplementary material for: Evaluating the effect of food components on the digestion of dietary nucleic acids in human gastric juice in vitro
Source: Food Sci Nutr. 2023 Aug 6;11(10):6522–31. doi: 10.1002/fsn3.3599 (PMC10563756; doi:10.1002/fsn3.3599)
Supplement: Supplementary file 1 — Figure S1. Figure S2. Figure S3. [file FSN3-11-6522-s001.pdf]

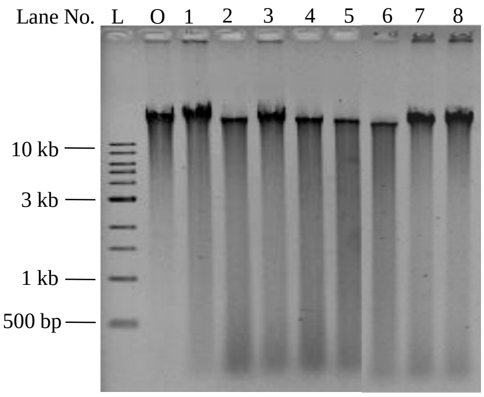

Supplementary Figure 1. Digestion of salmon sperm DNA *in vitro* by human gastric juice from eight individuals. The pH value from lane 1 to lane 8 was 2.6, 2.9, 2.3, 2.8, 1.7, 2.9, 1.6 and 2.3 respectively. Lane O, original salmon sperm DNA. All reactions were incubated at 37°C for 5 h.

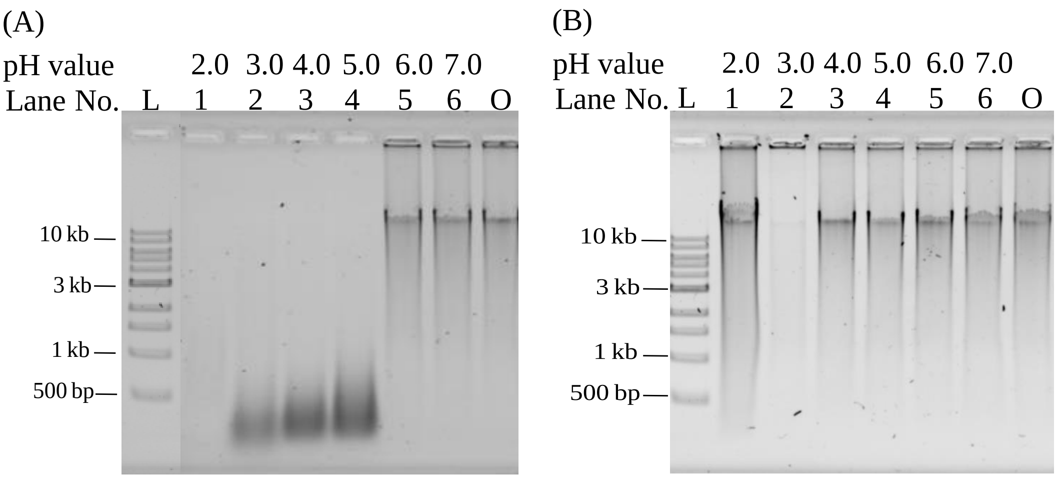

Supplementary Figure 2. Salmon sperm DNA was digested under various pH values with pepsin added (A) and without pepsin added (B), respectively. (A) DNA was digested by pepsin. Lane 1 to lane 5: DNA was digested by pepsin at pH 2.0, 3.0, 4.0, 5.0, 6.0 and 7.0 respectively. O, original salmon DNA. (B) DNA was digested without addition of pepsin. Lane 1 to lane 6: DNA was digested by pepsin at pH 2.0, 3.0, 4.0, 5.0, 6.0 and 7.0 respectively. O, original salmon DNA. Other conditions: reactions were incubated 37°C for 5 h with 4 mg/mL of pepsin, 25 mM NaH_2PO_4 and 15 mM NaCl.

（A）（B）

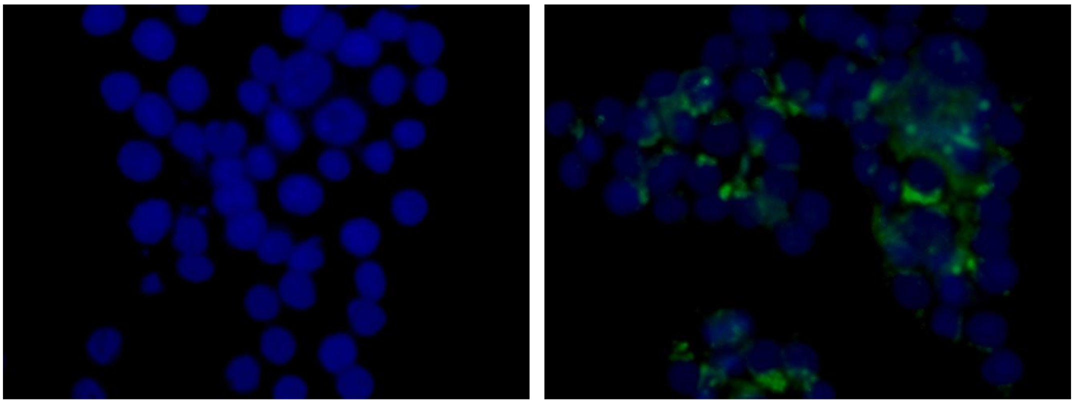

Supplementary Figure 3. Uptake of FITC-labeled 60 nt long single-stranded DNA by Caco-2 cells after 0 h (A) and 4 h (B) respectively. (×400.).
